# Nanotechnology for the Efficacious Delivery of Medicinal Cannabis and Pharmaceutical Medicines

**DOI:** 10.3390/ph18091385

**Published:** 2025-09-16

**Authors:** Luis Vitetta, Jeremy David Henson, Evan Hayes, David Rutolo, Sean Hall

**Affiliations:** 1 Department of Pharmacology, Sydney School of Pharmacy, Faculty of Medicine and Health, Camperdown Campus, The University of Sydney, Sydney, NSW 2060, Australia; 2 Company|Virile Group Level 24, 1 O’Connell Street, Sydney, NSW 2000, Australia; 3 Company|Medlab Group, 54 Royal Albert Cres, Paradise Point, QLD 4216, Australia; 4 Prince of Wales Clinical School, University of NSW, Sydney, NSW 2052, Australia

**Keywords:** nanotechnology, gastrointestinal tract, microbiota, microbiome, cannabinoids, nausea, pain, pharmaceutical drug delivery, NanoCelle^TM^, micelles, liposomes, dendrimers

## Abstract

The application of nanoparticles as nanomedicines, particularly for the targeted and efficacious delivery of drugs is an expanding platform in the field of cannabinoid and pharmaceutical drug delivery. By refocusing the route of drug administration beyond the oral gut pathway, this technology provides significant advancements that are especially relevant for cancer treatments. Orally administered drugs face significant challenges as they traverse the gastrointestinal tract (GIT) and are subject to first-pass GIT metabolism. Physiological conditions encountered in the GIT such as food effects, hormones, gastric pH, emptying time, and intestinal transit time vary widely across individuals. Fluid composition and enzymatic activity in the small intestine and large bowel also influence drug dissolution and absorption. These factors in conjunction with the intestinal cohort of bacteria can metabolize drugs before absorption, contributing to poor and variable drug bioavailability, which can be exacerbated by gut dysbiosis. Drug delivery that bypasses the oral-GIT route and hence first-pass metabolism offers a plausible solution for enhanced safety and drug efficacy.

## 1. Introduction

The oral administration of medicinal cannabis and pharmaceutical medicines remains the most patient-friendly route, offering both practical and psychological advantages, a route that generally preferred by both patients and healthcare providers [1,2]. Consequently, up to 90% of pharmaceutical compounds available for human use are formulated for oral delivery [2]. Oral delivery can significantly enhance patient adherence to treatment regimens, as it convenient, non-invasive, and allows patients to self-administer the drug. Orally administered drugs may be intended to exert their therapeutic effect locally within the GIT or be absorbed through the intestinal epithelium into the systemic circulation to reach distant target sites (e.g., the heart). Despite having widespread clinical preference, the oral route poses significant pharmacological challenges due to the GIT inherent complexity [3]. Oral-drug delivery complexity is subject to several factors, including the physiological variability of the GIT, enzymatic activity, pH fluctuations, and interactions with food and the intestinal microbiota [4]. Xenobiotic (e.g., antibiotics) modulation by intestinal bacteria can have detrimental effects on the gut microbiota that can lead to adverse drug effects and gut dysbiosis by promoting inflammatory sequalae in the gut [2].

Alternative modes of drug delivery can be historically traced to the 1960s when it was discovered that phospholipids when combined with water formed a sphere that began the nanotechnology revolution [5]. Nanoparticles and nanomaterials are progressively being investigated as delivery vehicles with potential applications in medicine.

Biochemically lipid nanoparticles, termed first generation liposomes, can be viewed as bilayer spheres formed and comprising one side of each molecule being water soluble, while the opposite side is insoluble in water [6,7,8]. Liposomes exhibit a broad size distribution, typically ranging from approximately 30 nanometers (nm) to several micrometers in diameter, depending on their method of preparation and intended application. Hence categorized as small (20–100 nm), large (100–1000 nm), or oversized (greater than 1000 nm) vesicles [9]. The specific size can influence their behavior, particularly in drug delivery applications [9,10] and represent versatile nanoplatforms for the enhanced transport of active natural compounds and pharmaceutical drugs in various matrices of biomedical and nanomedicine applications.

Nanotechnology comprises the science of bioengineering processes that leverage the manipulation of materials at the molecular scale for the elaboration and conversion of particulate matter into a physical state. This conversion produces nanoparticles of sizes of between 1 nm and 100 nm that can be reassembled into nano-systems with improved function [11]. Nanoparticles hold significance promise for the improvement of medical treatment specificity. It has often been posited that there is an extensive range in the doses administered of many drugs to produce a clinical efficacious result, given the pharmacological effects that have been observed in individual patients [12]. The individual patient variation in dose requirement is sometimes reflected in the wide scatter in the steady state plasma concentration that follows the same oral dose of a drug given to any group of patients [13].

Nanoparticles have been gaining prominence in drug delivery systems due to their capacity to enhance therapeutic efficacy while minimizing adverse effects. These carriers can be engineered for site-specific targeting, improved solubility and physicochemical stability of active pharmaceutical ingredients, and precise control over drug release kinetics. Such properties support the development of personalized treatment regimens with improved clinical outcomes [11]. Nanotechnology has significantly transformed modern medicine, particularly in the development of advanced drug delivery systems, utilizing both natural and synthetic compounds [14]. These nanocarriers enable precise delivery of therapeutics, enhancing efficacy while minimizing systemic toxicity. Due to their small size and tunable surface properties, many nanoparticles demonstrate enhanced pharmacokinetic behavior, including improved absorption, distribution, and retention. These characteristics enable the selective targeting of specific cell types, depending on the particle’s composition and functionalization [14]. Once internalized, nanoparticles can accumulate in the subcellular compartments of lysosomes, mitochondria, or the nucleus, with capabilities of modulating intracellular processes. This biochemical ability underpins a favorable therapeutic potential in managing chronic diseases, including diabetes, cancer, and kidney disorders or the management of symptoms such as pain and nausea. Nanoparticles can facilitate precise intracellular drug delivery and minimizing off-target effects [15].

Drug delivery can be achieved through various routes including, for example, intravenous (IV), intramuscular (IM), and oral administration, each with its advantages and disadvantages [16]. IV drug delivery entails direct administration into the bloodstream via needle insertion into a blood vessel, offering the most rapid onset and complete bioavailability of all delivery routes. In contrast, intramuscular (IM) injection leverages the rich vascular supply of muscle tissue to facilitate systemic absorption. Despite showing efficacy, both IV and IM modalities require needle penetration, often associated with discomfort and reduced patient acceptability [17].

We searched the medical and scientific literature from 2000 onwards for studies that have investigated specific nanotechnology platforms for the delivery of medical cannabis and pharmaceutical drugs. Therefore, we opted to review and adopt those nanotechnology platforms that utilized oro-buccal and or sublingual and or oral nanoparticle formulation for the delivery of pharmaceutical drugs and or cannabinoids. We posit that such delivery platforms present a plausible investigative idea for this narrative review that can overcome the disadvantages of oral administration of cannabinoids and pharmaceutical drugs that is encountered with first-pass metabolism in the intestines.

## 2. The Gastrointestinal Tract (GIT), Cannabinoids, and Pharmaceutical Drugs

### 2.1. The Intestinal Microbiome/Microbiota

**Figure 1:**
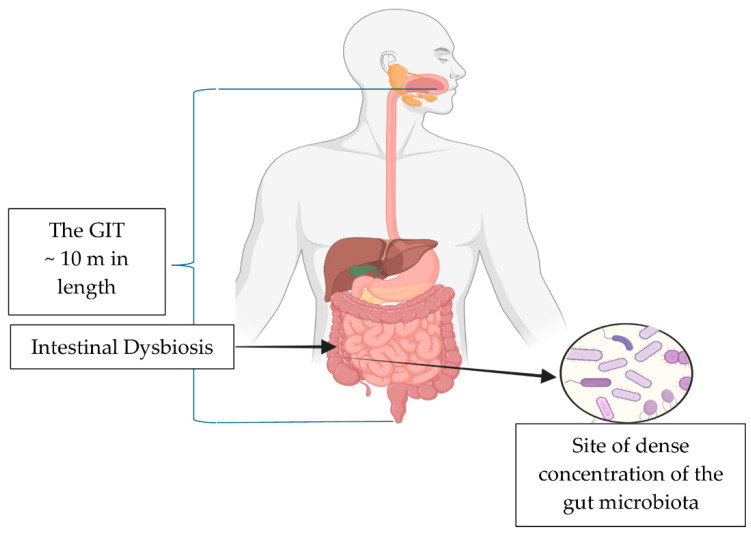
Diagrammatic representation of the GIT and the site of high concentrations of bacterial bioactivity in the ascending colon. Created in Biorender.com (accessed on 25 March 2025).

The GIT is an approximately 10 m long tube with an entry and exit [18] (Figure 1). A healthy gut is in a continuous state of equilibrium of pro- and anti-inflammatory responses. The microbiota in the intestines exerts a strong influence on the fluidity of all functions that occur daily, whether in the fed or fasted states [19]. The microbiota symbiosis that exists with the host governs health and disease states [19]. The combined microbiota–host symbiosis decisively directs and regulates the hematological structures (e.g., mucosal immunity) maintaining the equilibrium of the immune system. Moreover, the steady state also strengthens non-hematological structures (e.g., the intestinal epithelial barrier) by acting in concert to limit gut toxins/pathobionts translocation out of the gut lumen [19,20].

The adult human gut harbors a diverse microbial community composed of seven principal bacterial phyla, namely *Firmicutes* (including *Clostridium*, *Lactobacillus*, and *Enterococcus*), *Bacteroidetes* (e.g., *Bacteroides*), *Actinobacteria* (e.g., *Bifidobacterium*), *Proteobacteria* (e.g., *Escherichia coli*), *Fusobacteria, Verrucomicrobia*, and *Cyanobacteria.* The microbiota plays a fundamental role in maintaining homeostasis, influencing digestion, immunity, and systemic health [19]. Members of the *Firmicutes* and *Bacteroidetes* phyla are the dominant constituents of the human gut microbiota and have been implicated in promoting metabolic disturbances associated with obesity and colorectal cancer development [21].

The cohort of bacteria that inhabit the intestines represents an organ unto itself that has important correlations for nutrient absorption, by metabolizing foods for its own energy needs and for synthesizing for the host, essential beneficial vitamins, minerals, and amino acids for absorption and the detoxification/elimination of toxic food compounds produced [22]. The microbiota cohort in the gut represents a fundamental component of human physiology, exerting significant influence on both health and disease. This complex ecosystem, comprising bacteria, viruses, fungi, archaea, and helminths that inhabit the GIT, play a pivotal role in digestion, immune regulation, nutrient absorption, and the maintenance of mucosal integrity [23].

A recent comprehensive review on cannabis and microbiota interactions [24] identified several studies that demonstrate the impact of cannabis and cannabinoid on oral, gastrointestinal, fecal, and vaginal microbial abundance and diversity. Specific murine model studies have demonstrated the interactions that phytocannabinoids can have with the microbiota. For example, THC-attenuated lung inflammation altered the intestinal microbiota, enhancing the prevalence of *Ruminococcus gnavus* in the lung and the intestinal tissue of THC-treated mice, with a concomitant enrichment of short-chain fatty acids (SCFAs), particularly anti-inflammatory propionic acid [25]. An additional study observed that THC and CBD significantly reduced the clinical signs and high levels of the endotoxin lipopolysaccharide within the brains of mice with experimentally induced autoimmune encephalomyelitis [26]. This study also reported increases in anti-inflammatory cytokines and declines in pro-inflammatory cytokines, as well as reductions in the abundance of mucin-degrading *A. muciniphila* [26].

Moreover, the intestinal microbiota influences cannabinoid pharmacokinetics by metabolizing cannabinoids into active or inactive compounds [27]. This action influences the bioavailability and therapeutic effects that medicinal cannabis molecules may have. Intestinal bacteria have been reported to affect enzymes, such as β-glucuronidase, that can biochemically cleave the glucuronide conjugates of cannabinoids such as Δ9-THC, which then release the active form of the cannabis drug back into the systemic circulation [27].

Given that exogenous cannabinoids can interact as ligands with the endocannabinoid system of receptors, the intestinal microbiota influences the enterohepatic circulation of cannabinoids. Bile acids, which are chemically modified by the gut bacteria, form complexes with cannabinoids—an activity that leads to increased reabsorption and the prolonged retention of the compounds in the circulation and end organs, such as adipose tissue [28]. Consequently, intestinal microbiota-derived secondary bile acids from primary bile acids can go on to activate endocannabinoid receptors [29]. The changes that result from intestinal bacterial activity changes in endocannabinoid levels and cannabinoid receptor expression can determine various physiological processes [30].

The gut metabolites produced by the microbiota, particularly short-chain fatty acids (SCFAs) such as acetate, propionate, and butyrate, play a pivotal role in modulating the endocannabinoid system (ECS). The fermentation processes of dietary fiber by gut bacteria that produce SCFAs have been shown to influence ECS signaling pathways both locally within the gut and systemically. Through various interactions, SCFAs can modulate inflammation, gut permeability, and energy homeostasis, all of which are tightly regulated by the ECS. This crosstalk underscores a critical bidirectional relationship, whereby microbial activity can influence endocannabinoid tone, while ECS signaling can, in turn, shape the composition and function of the gut microbiota [31].

SCFAs, particularly butyrate, demonstrate potent anti-inflammatory properties within the GIT and display critical functions in maintaining the integrity of the intestinal epithelial barrier. Butyrate serves as a primary energy source for colonocytes and promotes the expression of tight junction proteins, thereby enhancing gut barrier permeability and preventing the translocation of pathogens and pro-inflammatory molecules into systemic circulation. SCFAs are produced by various microbiota resident members from the *Coprococcus*, *Roseburia*, *Facealibacterium, Bifidobacterium*, *Lactobacillus*, *Lachnospiraceae, Blautia*, and *Oscillospira* genera. It has been reported that butyrate can provide up to 70% of the energy requisite of intestinal epithelial cells [32]. This high energy demand is imperative for maintaining the gut epithelial cell’s tight-junction protein scaffold [32]. Additionally, butyrate exerts immunomodulatory effects by inhibiting histone deacetylases (HDACs) through the induced production of anti-inflammatory cytokines. Activities that lead to the suppression of pro-inflammatory gene expression and the induction of regulatory T cells (Tregs) further support immune homeostasis in the gut environment [32].

### 2.2. GIT Dysbiosis

An underestimated factor of importance in influencing drug metabolism, absorption, and efficacy is the intestinal resident microbiome [33]. The intestinal bacterial cohort that inhabits the gut can alter how drugs are processed, impacting efficacy [34] and influencing potential side effects [34]. This effect is progressed from the microbiota’s effect on enzymes that metabolize orally administered drugs [34]. The impact of the local gut biochemical activities on host drug-metabolizing enzymes is central in drug and nutrient absorption and immune system modulation [34].

Drug absorption is influenced by microbiota function. For example, tryptophan metabolites that gut bacteria elaborate are of critical importance for maintaining intestinal barrier mucosal integrity and permeability functionality [35]. Intestinal bacteria metabolizing tryptophan, an essential amino acid, gives rise to tryptophan bioactive molecules (e.g., indole and its derivatives) [36]. Several intestinal bacterial species have been reported to metabolize tryptophan to indole byproducts, including species such as *Bacteroides thetaiotaomicron*, *Proteus vulgaris* and *Pseudomonas aeruginosa* [37,38], and *Clostridium sporogenes* [39]. Importantly, tryptophan metabolites significantly influence the regulation of host mucosal immunity, actions that promote eubiosis (i.e., a balanced gut microbial cohort). Furthermore, gut bacterial metabolites can upregulate the expression of cannabinoid receptors (CB_1_ and CB_2_) as well as the enzymes (i.e., fatty acid amide hydrolase [FAAH] and monoacylglycerol lipase [MAGL]) that are involved in the synthesis and degradation turnover of endocannabinoids [40], provoking chemical reactions that may support cannabinoid ligand binding, thus influencing function and biological activity.

The dysbiosis of the intestinal refers to an imbalance in the composition and function of the gut microbiota [41]. The semi-permeable single cell lining of the intestinal barrier is a structure that covers the gut and prevents pathobionts themselves and the potentially pathogenic molecules that they can elaborate (e.g., lipopolysaccharides) from translocating across the gut mucosa and into the systemic circulation that, if breached through the effects that ensue from intestinal dysbiosis, can progress local inflammatory responses and systemic infections [22,41,42].

Dysbiosis, involving groups of bacteria in the gut and imbalances favoring *Firmicutes* over *Bacteroidetes*, has been linked to increased energy harvesting from the diet and pro-inflammatory states that may contribute to disease states [41]. Thus, the intestinal microbiota is influenced by a complex interplay of exogenous and endogenous factors [41]. The influence exerted can produce effects that range from transient shifts to enduring alterations in the intestinal microbial composition of gut species, such as decreased abundance of *F. prausnitzii* (i.e., exhibiting anti-inflammatory actions) with increased abundance of *C difficile* (e.g., exhibiting pro-inflammatory activities) with outcomes that span the spectrum from benign to pathogenic respectively [43,44].

Moreover, emerging evidence suggests that variations in specific gut bacterial families, including *Peptostreptococcaceae*, *Veillonellaceae*, and *Akkermansiaceae*, can influence the synthesis and degradation of endogenous cannabinoids. These microbial shifts may affect the levels and activity of key endocannabinoid compounds such as anandamide (AEA) and 2-arachidonoylglycerol (2-AG), thereby altering signaling through cannabinoid receptors in both peripheral and central tissues. This gut microbiota–endocannabinoid interaction is thought to play a role in regulating host metabolic processes, immune responses, and even mood and behavior [45]. Thus, the intestinal dysbiosis of the gut microbiota can disrupt the ECS, potentially impacting cannabinoid signaling, cannabinoid efficacy, and overall health.

Physiological conditions encountered in the GIT, such as food effects, hormones, gastric pH, emptying time, and intestinal transit time, vary widely across individuals and populations [46]. Fluid composition and enzymatic activity in the small intestine and colon also influencing drug dissolution and absorption. These factors in conjunction with the intestinal cohort of bacteria can metabolize drugs before absorption, contributing to drug bioavailability and with gut dysbiosis a further contributor to drug absorption and bioavailability [47]. What is critical is the transit time taken to pass through specific locations (e.g., stomach, small bowel, large bowel) of the GIT. Time to transit the GIT can affect drug dosage, especially for pharmaceuticals that are absorbed in specific regions or have region-specific actions (e.g., small bowel) [47]. Transit through the small intestine can range from 2 to 8 h with individual variations, whereas transit through the large bowel can vary between 6 and 80 h, whether in the fasted or fed state [46].

Circumventing the gastrointestinal tract and intestinal dysbiosis with nanotechnology delivery systems for medicinal cannabis and pharmaceutical medicines can result in enhanced drug efficacy. Reports advance the posit that oral delivered nanoparticles show promise in nanomedicine to improve drug delivery [48]. Engineering nanoparticles to overcome the harsh intestinal conditions and physical barriers that are encountered throughout the gut serves to achieve better systemic circulation and target tissues. Various strategies in the design of nanoparticles involve the penetration of the protective mucus layer, resist digestive enzymes and acidic stomach pH, and facilitate transport across the intestinal epithelial barrier [48]. The requisite for nanoparticle treatment efficacy should include shielding the therapeutic payload, protection from degradation, and improved absorption for the treatment of local gastrointestinal and systemic diseases.

## 3. Medicinal Cannabis

We refer the reader to the review by Legara and colleagues [49], where the authors reviewed the therapeutic potential of medicinal cannabis molecules for the treatment of various disease states. The review presents data for pure medicinal cannabis compounds (e.g., dronabinol, nabilone, and CBD), as well as partially purified medicinal cannabis extracts (e.g., nabiximols), to provide guidance on the potential therapeutic uses of high-THC cannabis and CBD orally delivered oils for numerous conditions [49].

Furthermore, a recent review has investigated studies that portend to provide biochemical evidence on the effect that cannabinoids may have on cellular signaling pathways, namely those associated with reactive oxygen species, AMP-activated protein kinase, mitogen-activated protein kinases, phosphoinositide 3-kinase, hypoxia-inducible factor-1alpha, and p53 [50]. The authors posit that cannabinoids could mediate the reprogramming of cancer metabolism such as glucose metabolism, lipid metabolism, and amino acid metabolism [50]. This aspect of cellular metabolism is of utmost importance given that the modulation of cancer metabolism is currently an active area of cancer treatments.

Thus, in this section, we present a synopsis of the administration of medicinal cannabis products for medical disorders for which cannabis has the highest level of clinical evidence, and these include for the treatment of nausea, pain, seizures, appetite stimulation, and muscle spasticity.

### 3.1. Medicinal Cannabis for Nausea

Despite advancements in anti-emetic pharmacotherapy and associated nausea, which includes the development of serotonin 5-HT3 and neurokinin-1 (NK1) receptor antagonists, chemotherapy-induced nausea and vomiting (CINV) remains a significant clinical challenge. As so does anticipatory nausea and vomiting in patients expecting to undergo chemotherapeutic treatments [51]. This adverse effect not only impairs patients’ ability to complete chemotherapy regimens but also ranks among the most distressing complications of cancer treatment. CINV has been associated with a decline in quality of life, as well as deterioration in both physical and cognitive functioning, which may ultimately impact a patient’s willingness to continue therapy.

Cannabinoids, known for their wide-ranging physiological effects on the nervous, immune, cardiovascular, and gastrointestinal systems, have been proposed as alternative and potential useful therapeutic agents for the treatment of nausea and vomiting [52]. An early systematic review and meta-analysis examining the efficacy of synthetic cannabinoids, such as dronabinol, nabilone, and levonantradol, for CINV included 30 clinical studies, some of which compared these agents with placebo, while others used conventional anti-emetics as comparators [53]. Although limitations related to small sample sizes and heterogeneity in study designs prevent definitive conclusions, available data suggest that dronabinol may be more effective as an acute anti-emetic than some standard anti-emetic therapies.

A small randomized, double-blind study also reported that nabiximols (a standardized cannabis extract, containing tetrahydrocannabinol and cannabidiol as its primary active components), when used as an adjunct to standard anti-emetic regimens, demonstrated efficacy in managing CINV [54]. Anecdotally, many cancer patients have expressed a preference for smoked cannabis over synthetic cannabinoids, citing superior anti-emetic effects [55]. However, this observation lacks support from controlled clinical trials. Consequently, cannabinoid therapies are not routinely integrated into oncology protocols, in part due to the absence of standardized formulations, defined dosing strategies, and comprehensive data on patient tolerability in this context. Nevertheless, cannabinoids have a potential major role in managing an array of gastrointestinal conditions [40] if delivered via other modalities that avoid the extensive first-pass metabolism that cannabinoids experience when absorbed though the GIT.

### 3.2. Medicinal Cannabis for Pain

Relief from chronic pain either from cancer or non-cancer-related diseases is a commonly cited by patients who express a desire to administer medicinal cannabis [56,57]. An extensive meta-analytic review of the literature consisting of 43 randomized controlled trials (RCTs) reported a feasible posit for the administration of medicinal cannabis-based-medicines to effectively improve chronic pain treatment and primarily for neuropathic pain [58]. Additional reports have postulated that cannabinoid-based pharmacotherapies may serve as effective replacement/adjunctive analgesic options [59].

Neuropathic pain has been the most common form of pain investigated with the administration of medicinal cannabis products to induce an analgesic effect. Investigations have reported that low dose Delta-9-tetrahydrocannabinol (Δ9-THC) (1.29%) as vaporized cannabis was superior for relieving central or peripheral neuropathic pain that had been shown to be resistant to standard treatments when compared to placebo [60]. Additional studies that examined the oral/oro-mucosal routes for medicinal cannabis administration, often as crude herbal or dry leaf cannabis extracts, as synthetic versions of THC (e.g., dronabinol, nabilone), or as plant-derived extracts of THC/CBD (i.e., oromucosal spray, nabiximols), have shown limited efficacy in treating chronic neuropathic pain [57]. When all medicinal cannabis-based-medicines were pooled, they were better than placebo in reducing problems associated with sleep disturbances and concomitantly improved psychological distress and health-related quality of life [57]. Medicinal cannabis may also be effective for other types of pain (e.g., nociceptive pain, nociplastic pain). Clinical studies administering smoked cannabis extracts to patients diagnosed with postsurgical or post-traumatic pain [61] and to those with painful human immunodeficiency virus (HIV)-associated neuropathy [62,63,64] all reported the efficacy over placebo in terms of the relief of pain, with good tolerability to the medicinal cannabis used [64].

Although, the effectiveness of medicinal cannabis for chronic pain has been established [65], all five previous clinical trials of medicinal cannabis for cancer pain (as reviewed by Boland and colleagues [66]) have failed to reach the efficacy endpoint. The oral administration of cannabis-based medicines with gastrointestinal absorption leads to the highly variable systemic concentrations of pharmacologically active constituents, resulting in slow and erratic onsets of action for analgesic use [67]. Frequent dosing is required for inhaled cannabis to maintain an analgesic effect given that there is reported a half-life of less than 20 min [67,68]. Furthermore, high THC blood concentrations (20-fold to 30-fold higher than C_max_) after inhalation administration is associated with treatment limiting acute adverse effects and possible long-term respiratory system damage from toxic chemicals associated with smoking or vaporizing with a medicinal cannabis product [67] (high temperatures involved with vaporized cannabis can oxidize the medicine and excipients) [69].

Oral and inhaled administration routes for medicinal cannabis extracts presents a clinical picture that is unsatisfactory for the management of pain, and this has led to the additional proposed routes of administration such as transdermal, transmucosal, and intranasal modes of delivery [70]. In addition, due to their lipophilic nature, cannabinoids show promise as highly regulated prescribed medicines [70] and continued investigations on the implementation of nanoparticle delivery technologies are needed.

### 3.3. Medicinal Cannabis for Seizures

A chronic condition of recurrent seizures often presents in childhood and arises out of abnormal excitation–inhibition balance of neurons in the brain. It has been posited that cannabinoids could act as neuroprotective agents reducing inflammatory responses in patients diagnosed with epilepsy [71,72,73]. Clinically, CBD has been reported to have a higher enhanced efficacy than that provided by whole-plant cannabis extracts for the treatment of epileptic seizures [73]. Case reports and surveys have described smoked cannabis as having both pro- and anti-convulsant properties [73]. Open label clinical studies on patients with treatment-resistant epilepsy who received doses of oral CBD for 3 months as an adjunctive therapy to their current anti-epileptic medications reported efficacy with an approximately 50% responder rate and a reduction in weekly seizure frequency of just under 50% [74,75]. Irrespective of the reported variations in patients inducted into these studies, the results were mirrored in patients with seizures caused by conditions such as Dravet syndrome and tuberous sclerosis complex [75]. In 2018, CBD branded as Epidyolex^®^ received FDA approval for the treatment of selected intractable seizure disorders in children, with approval from the EMA following in 2019.

### 3.4. Medicinal Cannabis for Appetite

A systematic review on medicinal cannabis extracts and appetite have shown promise in improving appetite-related symptoms in people with cancer [76]. Five studies have reported on the administration of medicinal cannabis extracts for the management of anorexia that informs on the loss of appetite in people diagnosed with cancer [76]. Anorexia is a prevalent and distressing symptom with limited effective interventions, and therefore investigating medicinal cannabis efficacy in this clinical scenario is an important research topic [76].

Four studies used a placebo as a comparator [77,78,79,80], while the fifth study used megestrol acetate [81]. The results from these five clinical studies present an overall contentious picture of efficacy to manage cancer-related anorexia and appetite. That is, THC may be useful in the palliation of chemosensory alterations and to improve food enjoyment for cancer patients [77]. Other studies reported no differences in patients’ appetite or QOL [78]. In a different study, nabilone was reported to not be potent enough to improve the patients’ quality of life when compared to placebo [79]. Nabilone was also investigated, and the study concluded that it was an adequate and safe therapeutic option to aid in the treatment of patients diagnosed with anorexia [80].

In patients with anorexia resulting from cancer, there are many complex symptoms to consider with numerous potential mechanisms. From the paucity of clinical studies that are currently available, results on the use of medicinal cannabis to treat appetite due to cancer-induced anorexia should be treated with caution.

### 3.5. Medicinal Cannabis for Muscle Spasticity

In multiple sclerosis, a progressive demyelinating disease, the observation that multiple sclerosis exhibits changes in the expression of CB_1_ and CB_2_ receptors may provide early evidence for the therapeutic benefits of administering medicinal cannabis [82]. Sativex, a mouth spray containing Δ9-THC plus CBD, has been approved in several countries for the treatment of spasticity associated with multiple sclerosis [83]. It is interesting to note that Δ9-THC alone was found to be ineffective in the management of multiple sclerosis in a multi-year CUPID study [84]. Moreover, in addition to spasticity, treatment with nabiximols has also been reported to be beneficially effective in alleviating multiple sclerosis-induced pain [85,86].

## 4. Nanotechnology for Effective Cannabinoid and Pharmaceutical Drug Delivery 

The implementation of nanotechnologies has transformed innovations in medicine, specifically in diagnostic methods, imaging, and, importantly, in pharmaceutical drug delivery [87]. Several platforms have been developed to deliver medicines using alternatives to the oral-GIT route [88,89]. Liu and colleagues [90] recently advanced the idea of the construction of nanomedicines using the active ingredients of natural products (e.g., cannabinoids), giving rise to key steps in research on innovative delivery platforms. They highlight the improvements that natural product nanotechnologies may provide, such as overcoming issues of low solubility, large dosages, poor bioavailability, and poor targeting [90]. Nanotechnologies are posited as enhancing the safety, selectivity, and efficacy of natural products, thus elevating natural product-based nanomedicines as promising candidates in clinical medicine [90].

In a recent review Singh and colleagues [91] analyzed several studies and patents that outlined how nanomedicine medical cannabis formulations can provide potential therapeutic benefits for managing pain, anxiety, depression, and neurological and movement disorders. The review also highlighted the synthesis of studies that expand the potential of nanotechnology to enhance cannabis therapies [91]. Specifically, Singh et al. [91], when analyzing 7 of 36 studies, reported that nanoparticle technologies applied to medicinal cannabis molecules provided enhanced cannabinoid bioavailability, while other publications focused on better cannabinoid stability, payload release, and solubility. In 7 of 19 patents also examined by Singh et al. [91], enhanced bioavailability was reported, while the remaining patents covered various formulation methods. Overall, the solutions that the authors investigated in their review were suggested to possibly lead to innovative interventions for precision medicine [91,92].

The following sections outline examples of nanotechnologies developed specifically as platforms for the delivery of pharmaceutical medicines and for medicinal cannabis molecules (Table 1).

### 4.1. Micelles

Micelles are nanoscale aggregates composed of amphiphilic surfactant molecules, typically including lipids, that self-assemble under aqueous conditions. These structures form spherical vesicles characterized by a hydrophilic outer monolayer and a hydrophobic core, enabling the encapsulation of hydrophobic therapeutic agents. The amphiphilic nature of micelles significantly enhances the aqueous solubility of poorly water-soluble drugs, thereby improving their bioavailability. Micelles can typically range in diameter from 10 to 100 nm. Owing to their unique physicochemical properties, micelles have been extensively utilized as drug delivery systems with therapeutic applications [89].

The continued development of high-quality medicinal cannabis plant extracts and synthetic cannabis formulations [101,102], such as nabiximols (Sativex^®^, a cannabis extract with equimolar THC and CBD developed by GW Pharmaceuticals), have been granted approval (e.g., FDA) for use in multiple sclerosis-associated inflammatory spasticity in multiple locations. The oro-mucosal mouth spray, using 50% ethanol, delivers 2.7 mg THC and 2.5 mg CBD from *Cannabis sativa* L. with each 100 µL actuation. Furthermore, an additional formulation of an oral CBD solution Epidyolex^®^ (also from GW Pharmaceuticals, subsequently acquired by Jazz Pharmaceuticals in 2021), labeled an orphan drug by the FDA, has also been approved and indicated for the treatment of seizures associated with Lennox–Gastaut syndrome in both adults and pediatric patients, with the latter being older than two years of age. Regulatory agencies maintain that an important clinical requisite is that clinical studies must present patient data with correct and accurate standardized doses that were delivered to achieve optimal therapeutic efficacy [103].

**Figure 2:**
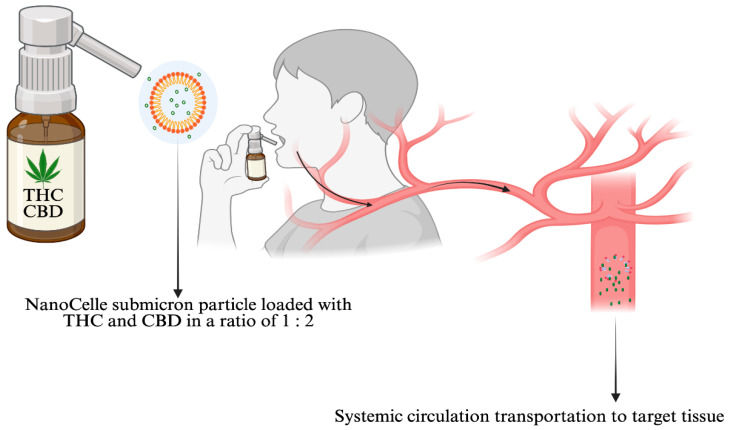
NanoCelle^TM^ technology: the oro-buccal delivery of medicinal cannabis through the facial lymphatics and from there into the systemic circulation, transported to target tissues [Created in BioRender (https://BioRender.com), Vitetta, L. accessed on 27 May 2025].

NanoCelle^TM^ is an innovative submicron particle delivery platform for the oral mucous membrane absorption of small lipid soluble molecules, such as THC and CBD [93] or other pharmaceutical medicines that adhere to the chemical properties attributed to micelles. NanoCelle™ is a self-assembled, micellized nanoparticle drug delivery system designed for administration via the oro-buccal mucosa [89]. Comprising a hydrophobic core and hydrophilic shell, NanoCelle™ particles facilitate the passive diffusion of small molecules, such as vitamins (e.g., B12), across the oral mucosal membrane [104] (Figure 2). The technology has been extended to deliver cannabinoids [105,106], and early clinical trials have demonstrated a favorable safety profile and consistent pharmacokinetics [93]. Following published in vitro liposome investigations on red algae [107], we also conducted in vitro experiments with NanoCelle^TM^ formulations encapsulating THC and CBD combined and CBD alone (personal communication, i.e., unpublished data). The characterizations of the micelle formulations demonstrated mean particle sizes of 33 nm (THC + CBD) and 20 nm (CBD), respectively, determined by Zetasizer.

Micellar nanoparticles have also been developed for drug administration [11]. The intravenous injection of a micellar containing paclitaxel was FDA-approved for the treatment of several cancers, including breast, non-small cell lung, pancreatic cancer, and ovarian cancers [92]. Recent advances in micellar nanoparticles have been suggested as being suitable for cardiovascular targets of diagnosis and treatments [108]. In addition, nanotechnology-based drug delivery platforms are being developed for the treatment of erectile dysfunction [109].

Micellar nanoparticles enabled the production of clear, stable aqueous solutions of relatively water-insoluble drug components without altering their chemical structures. This provides flexibility that allows the development of aqueous nanoparticle formulations of oro-mucosal, nasal, ocular, and transdermal products without the use of alcohol or the first-pass passage and metabolism through the GIT.

### 4.2. Liposomes

Liposomes are spherical vesicles composed of lipid bilayers, with particle sizes typically ranging from 30 nm to several micrometers. They possess the unique capability to encapsulate both hydrophilic therapeutic agents within their aqueous core and hydrophobic agents within the lipid bilayer structure. Due to their structural versatility, liposomes can be surface-modified with polymers, antibodies, and proteins, enabling the incorporation of macromolecular therapeutics, such as nucleic acids and crystalline metals [11]. A commonly cited example is poly(ethylene glycol) (PEG)-ylated liposomal doxorubicin (Doxil^®^), listed as the first FDA-approved nanomedicine. This approved liposomal drug is employed in the treatment of breast cancer, enhancing drug accumulation within malignant effusions while avoiding an increase in systemic dosage. In addition, liposomes with dedicated uses as drug delivery systems can sometimes trigger an adverse and severe immune response, such as complement activation-related pseudoallergy (CARPA) [110,111]. This reaction, characterized by hypersensitivity symptoms, is not a true allergic reaction mediated by IgE antibodies, but is an immune response mediated by the complement system.

Numerous nanodrugs have been approved by the FDA for clinical applications [11]. Examples include pharmaceuticals for intravenous injections (e.g., amphotericin B) [112], intramuscular/intrathecal/subcutaneous injections (e.g., daunorubicin) [113], and specific subcutaneous injections (e.g., Pegylated IFN-beta-1a) [92].

### 4.3. Dendrimers

Dendrimers exhibit a size of less than 100 nm and a branched three-dimensional structure of repeating units, extending from a centric core and comprising peripheral functional groups [87]. Dendrimers encapsulate therapeutic agents as other nanoparticle structures do, within the interior space consisting of the formed dendrimers. Moreover, the therapeutic agents can be attached to surface groups, making dendrimers highly bioavailable and decomposable. Chemical conjugates of dendrimers have been shown to exhibit enhanced antimicrobial, antiprion, and antiviral properties when they encapsulate peptides or saccharides, showing improved solubility and stability on the absorption of the therapeutic drugs [114].

Dendrimer-based drug delivery formulations that involve poly(amidoamine) and proton-pump-inhibitor dendrimers conjugated with drugs like methotrexate, paclitaxel, and doxorubicin for cancer therapy and VivaGel™ as a vaginal microbicide are also under investigation [115].

## 5. Discussion

The nanotechnology revolution has profoundly influenced nearly every sector of modern society, offering innovative solutions that are better engineered, safer, more sustainable, longer lasting, and increasingly efficacious for the delivery of medicines. 

The integration of nanomaterials into products is generally carried out via two main approaches. Firstly, nanoparticles are incorporated into existing materials to enhance composite performance through the unique physicochemical properties of nanoscale additives. Secondly, nanomaterials, such as nanoparticles or nanocrystals, are directly used to construct novel high-performance devices [11]. These scientific advancements have the potential to redefine the capabilities of medical and industrial applications and shape the future of technology, especially in the delivery of medicines with the aim of enhancing safety and efficacy of medical treatments.

We have recently shown that intestinal dysbiosis can significantly affect the absorption of cannabinoids (e.g., cannabidiol [CDB]) [116]. CBD is poorly water soluble, and ingestion via the gut provides poor absorption [117]; most of the CBD that is absorbed undergoes first-pass metabolism, resulting in a very low bioavailability of about 6% [117]. Systemic exposure to CBD is increased four-fold through ingestion with a high-fat meal [86] and five-fold with severe hepatic impairment [118]. The physiology of the intestines is subject to increased absorption with a high-fat meal because of the formation of bile salt micelles, which are naturally formed in the intestines through the action of bacteria admixed with a high-fat meal or orally delivered medicinal cannabis products (e.g., oil-based formulation CBD) [116]. Thus, the largely naturally formed micelles can transport CBD molecules from the intestinal epithelial cells across to the portal/systemic circulation [116]. Thus, as products containing CBD for administration as sublingual oil-based drops still result in the first-pass metabolites of CBD are ineffective, this indicates that mucous membrane absorption is inefficient [119].

A proposed alternative is inhaling CBD by smoking or vaping, which is known to be a rapid method of CBD administration, reflecting a time to peak plasma concentration of less than 5 min and a bioavailability of 31% [120]. However, it is accepted that the highest level of CBD inhalation that can be achieved is associated with increased adverse effects, and the high temperatures required to inhale cannabinoids increases the likelihood of toxic oxidation products produced and inhaled [119] by the lungs [67]. Thus, it is feasible to postulate with our current understanding of nanotechnologies that oral mucous membrane absorption could be improved by mimicking the carriage of CBD in natural intestinal micelles. Formulating CBD in synthetic nanoparticle matrices and delivering a proposed drug or the cannabinoid CDB pre-formed as a synthetic nanoparticle across the oro-buccal mucous membrane instead of the intestinal mucous membrane avoids first-pass metabolism in the gut and enhances efficacy [106]. The oro-buccal delivery of drugs across a mucosal membrane may constitute a better transport platform for enhanced drug delivery efficacy with reduced side effects.

Nanomedicine and nanocarrier-linked medical approaches are in a rapid flux of development through the science that leverages the biomolecular nanoscale range. These contributions to the site-specific delivery of medicines in a controlled manner have resulted in considerable research interest, mainly regarding their potential to enhance bioavailability, decrease adverse side effects, and avoid first-pass metabolism in the intestines, which is critically important. This is especially pertinent to the delivery of cannabinoids and pharmaceutical medicines though the oral–gut route that limits absorption. Therefore, novel approaches that deliver cannabinoids and pharmaceutical medicines via an oral–buccal delivery water-soluble matrix have been shown by modeling studies to provide enhanced bioavailability [93]. Technologies that provide evidence to support the application of innovative drug delivery platforms (e.g., micellar, dendrimers) to overcome limitations associated with cannabinoid administration via the oral–gut route provide significant credibility for therapeutic use.

## 6. Conclusions

Bypassing the first-pass metabolism in the gut is a fundamental and important characteristic of nanomedicines. It is thus possible to identify nanoparticles that form clear solutions in a stable aqueous matrix. Producing relatively insoluble drug components without altering their chemical structures is an important feature of nanomedicine drug delivery platforms. These nanomedicines provide flexibility that allows the development of nanoparticle aqueous formulations of oro-mucosal, nasal, ocular, and transdermal products without the use of alcohol for enhanced delivery which bypasses the first-pass passage and metabolism of the GIT.

## Figures and Tables

**Table 1:** Nanoparticle delivery platforms and examples of pharmaceutical drugs and medicinal cannabis administration.

Drug Manufacturer Brand/Trade Name [Ref]	Pharmaceutical	Method of Delivery	Indication	Advantages and Disadvantages
**MICELLES** **Usual size range 5 to 100 nm|surface charge can be anionic|cationic|zwitterionic**				
Celgene, Summit, NJ, USA Abraxane [92]	Paclitaxel	Intravenous Administration	-Breast cancer -Non-Small Lung cancer -Pancreatic cancer -Ovarian cancer	Advantages ↑drug solubility| Nanosize prolonged circulation|Hydrophilic shells| Improved bioavailability of hydrophobic drugs| Disadvantages Drug loading efficiency|Difficulty controlling particle uniformity| Premature drug release|Instability in physiological environments| Potential immunogenicity or toxicity issues|
Medlab Group, Paradise Point, QLD, Australia MDCNB-01 (THC + CBD) [93]	MDCNB-01 (NanaBis)	Orobuccal Administration	-Cancer Pain -Cancer Nausea	
Zentopia, Boise, ID, USA Pink Lemon Smash THC + CBD [www.Zentopia.com]	Pink Lemon Smash	Oral liquid	-General consumption	
Vacay, Valens Agritech Ltd., Kelowna, BC, Canada Vacay’s Island Punch [https://www.vacayedibles.com]	Vacay’s Island Punch	Oral liquid	-General consumption	
**LIPOSOMES** **Usual size range 25 nm to 2.5 μm|surface charges can be neutral, negative, or positive**				
Pacira Pharmaceuticals, San Diego, CA, USA DepoDur [94]	Morphine injectable (slow release)	Epidural Administration	-Postoperative pain	Advantages ↑ Drug stability| ↑ Targeted delivery| ↑ Bioavailability| ↓Toxicity| Biocompatible|Biodegradable nature ideal for delivering drugs and genes to specific tissues Disadvantages High production costs| Poor physical stability| Short circulation times Potential leakage or fusion of encapsulated substance|Chemical degradation of phospholipids|Instability in physiological environments| Potential immunogenicity or toxicity issues|
Marqibo (Spectrum Pharmaceuticals), Boston, MA, USA Oncovin|Vincasar PFS|Vincrex [95]	Vincristine	Intravenous Infusion	-Acute Lymphoblastic Leukemia	
Ipsen Biopharmaceuticals, Paris, France Onivyde [96]	Irinotecan	Intravenous Injection	-Metastatic Adenocarcinoma of the Pancreas	
Jazz Pharmaceuticals, Dublin, Ireland Vyxeos [97]	Daunorubicin and Cytarabine	-Intramuscular -Intrathecal -Subcutaneous Injection	-Acute Myeloid Leukemia	
Creative Biostructure, Shirley, NY, USA Lipo-308C [CBD] *Cannabis sativa* extract	Liposome for cosmetics| Liposomes for food	Sprays Oils	-General consumption	
**DENDRIMERS (polymer nanoparticles)** **Usual size range 1 to 100 nm|positive surface charge for cell entry acquisition**				
Tolmar, Chicago, IL, USA Eligard [98]	Leuprolide acetate and polymer	Subcutaneous Injections	-Prostate cancer	Advantages Precise drug delivery| ↑ Solubility| ↑ Targeting due unique branched monodisperse structure and customizable surface| Disadvantages High manufacturing costs|Potential toxicity issues [e.g., cationic dendrimers] Challenges in achieving pure and large-scale synthesis|
Pfizer, New York, NY, USA Somavert [99]	Pegvisomant	Subcutaneous Injections	-Acromegaly	
UCB, Brussels, Belgium Cimzia [100]	Certolizumab	Tablets or Intravenous Injections	-Rheumatoid Arthritis -Crohn’s Disease -Psoriatic Arthritis, -Ankylosing Spondylitis	

## Data Availability

Not applicable.
